# A High-Performance FPGA PRNG Based on Multiple Deep-Dynamic Transformations

**DOI:** 10.3390/e26080671

**Published:** 2024-08-07

**Authors:** Shouliang Li, Zichen Lin, Yi Yang, Ruixuan Ning

**Affiliations:** School of Information Science & Engineering, Lanzhou University, Lanzhou 730000, China; lishoul@lzu.edu.cn (S.L.); linzch21@lzu.edu.cn (Z.L.); yy@lzu.edu.cn (Y.Y.)

**Keywords:** random number generator, chaotic map, FPGA implementation, multiple deep-dynamic transformation (MDDT), pseudo-random number generator (PRNG), NIST SP800-22, diehard tests, TestU01, high-speed computing, embedded systems, cybersecurity, nonlinear dynamics

## Abstract

Pseudo-random number generators (PRNGs) are important cornerstones of many fields, such as statistical analysis and cryptography, and the need for PRNGs for information security (in fields such as blockchain, big data, and artificial intelligence) is becoming increasingly prominent, resulting in a steadily growing demand for high-speed, high-quality random number generators. To meet this demand, the multiple deep-dynamic transformation (MDDT) algorithm is innovatively developed. This algorithm is incorporated into the skewed tent map, endowing it with more complex dynamical properties. The improved one-dimensional discrete chaotic mapping method is effectively realized on a field-programmable gate array (FPGA), specifically the Xilinx xc7k325tffg900-2 model. The proposed pseudo-random number generator (PRNG) successfully passes all evaluations of the National Institute of Standards and Technology (NIST) SP800-22, diehard, and TestU01 test suites. Additional experimental results show that the PRNG, possessing high novelty performance, operates efficiently at a clock frequency of 150 MHz, achieving a maximum throughput of 14.4 Gbps. This performance not only surpasses that of most related studies but also makes it exceptionally suitable for embedded applications.

## 1. Introduction

Random number generators (RNGs) are systems that generate sequences of numbers that exhibit statistical independence and lack correlation, achieved through hardware and software methods. RNGs are classified into three primary types: pseudo-random number generators (PRNGs), true random number generators (TRNGs), and hybrid random number generators (HRNGs) [1,2,3]. These random numbers are essential in various applications, including computer simulations, numerical analysis, statistical analysis, Monte Carlo methods, IoT security, and cryptography [4,5,6,7].

As one of the aforementioned types of RNGs, pseudo-random number generators (PRNGs) are commonly utilized in the field of cryptography and contemporary security engineering, appreciated for their straightforward implementation and cost efficiency. Embedded systems, especially those integrated into the Internet of Things (IoT), depend on PRNGs for securing data through encryption. At the same time, devices in these application scenarios often face stringent resource and energy constraints, making the development of a fast and low-power-consumption PRNG particularly important. A low resource-consumption PRNG can effectively reduce the energy consumption of a device and extend battery life while maintaining a high operating efficiency, which is essential to ensure the reliability and efficiency of systems that run for long periods of time. To generate pseudo-random numbers, chaotic systems are often employed in PRNGs due to their complex behaviors that originate from deterministic processes, including recursive functions and sensitivity to initial conditions, enhancing the randomness of outputs. Diverse chaotic maps, such as logistic maps, Bernoulli shift maps, tent maps, and zigzag maps, are employed to enhance PRNGs, each contributing a distinct level of randomness and intricacy. However, the digital implementation of these chaotic systems frequently culminates in pseudo-chaotic orbits characterized by brief periods and notable correlations, primarily due to the limitations of finite precision and rounding errors present in both fixed-point and floating-point arithmetic.

Despite their deterministic nature, PRNGs are widely adopted in cryptography and other security-related fields because they are straightforward to implement and inexpensive to generate. They are used for generating and distributing encryption keys, creating initialization vectors, producing prime numbers and passwords, preventing side-channel attacks, and facilitating authentication protocols. Although PRNGs are finite state machines driven by deterministic algorithms, which means their outputs are not entirely random, their practicality and low cost make them advantageous. However, deterministic algorithms mean that if the algorithm is known, subsequent outputs can be predicted based on any given state, which limits their use in cryptographic algorithms that need to ensure unpredictability and security [8,9,10].

To enhance the randomness and security of PRNGs, chaotic systems are utilized not only as initial seeds but also as dynamic entropy sources throughout the generation process. The inherent sensitivity to initial conditions and unpredictability of chaotic systems make them excellent candidates for improving PRNG performance. Additionally, chaotic systems are characterized by low power consumption and high-frequency operation, making them particularly suitable for chaotic PRNG research using ASIC/FPGA technologies [11,12,13].

In the realm of pseudorandom number generator (PRNG) development, numerous researchers have historically leveraged chaotic maps to achieve significant success across various evaluations. Kocarev et al. pioneered a PRNG design utilizing chaotic maps that met all international standards [4]. Merah et al. advanced this field by creating an FPGA-based PRNG employing the Chua chaotic system, which passed all tests and exhibited promising applications in image encryption [14]. Furthering these efforts, Avaroglu et al. developed a hybrid PRNG that enhanced an AES-based structure with a chaotic 3D Sprott 94 G system implemented on an FPGA [15]. Meranza-Castillón et al. proposed an FPGA-based Henon map chaotic PRNG, which passed all statistical tests and achieved a throughput of 9 Mbps, demonstrating its viability in chaotic software/hardware encryption applications [12]. Rezk et al. explored PRNG designs based on 3D Lorenz and Lü chaotic systems on the FPGA platform, successfully passing all NIST statistical tests [16]. Patidar et al. introduced an innovative PRNG structure based on a chaotic map that excelled in both NIST and diehard tests [17]. Ahadpour et al. unveiled a new PRNG design rooted in chaotic logistics, passing all evaluations [18]. Elmanfaloty et al. examined PRNG designs based on 1D chaotic systems, which also met all international testing standards [19].

Despite the successes of these studies in various tests, limitations remain regarding period, throughput, and resource utilization. Recent research highlights these constraints: Luis Gerardo de la Fraga [20] proposed a Brownian system-based method with adequate occupancy for embedded development but limited throughput to 1085 Mb/s. Yinzhe Liu developed a random number algorithm based on the discrete chaotic system method, reaching a throughput of 10 Gb/s, but with high computational unit consumption, using 4694 LUTs [21]. In comparison, our approach achieves a throughput that is more than 1.4 times that of what Liu et al. reported, with lower resource occupancy.

In this study, we develop a random number generator (RNG) based on chaotic mapping with multiple deep-dynamic transformations. This multiple deep-dynamic transformation (MDDT) algorithm, first proposed by us, goes beyond the traditional tilted tent mapping by replacing fixed division operations with dynamic shifts and basic arithmetic operations such as addition, subtraction, and XOR. This innovation significantly simplifies the complexities associated with traditional multiplication and division operations. Furthermore, in the FPGA implementation, chaotic maps do not employ multiplication or division operations, which results in hardware utilization remaining highly competitive, consuming less than 1% of total resources. This level of hardware consumption not only retains a competitive edge in FPGA implementation but also ensures an impressive throughput of 14.4 Gbps/s, which allows our PRNG to stand out in a field crowded with numerous studies. This achievement unequivocally demonstrates the superior performance of our design. The PRNG implementation has successfully passed the most famous international benchmarks, including those set by the National Institute of Standards and Technology (NIST) SP800-22, diehard, and TestU01 test suites. This validates the effectiveness of the design.

The organization of this paper is as follows: We first outline the prevalent challenges in modern pseudo-random number generation and subsequently highlight the advantages of chaotic systems. Subsequently, in the ensuing chapters, we expound upon our theoretical algorithm, incorporating dynamical analysis and parameter scrutiny. The fourth chapter is dedicated to showcasing the algorithm’s implementation and performance optimization on FPGA. Moving forward, in the fifth chapter, we validate the efficacy of our design through experimental validation and comparative analysis vis-à-vis alternative methodologies. Finally, we engage in a discourse regarding the advantages inherent in our proposed algorithm and offer a succinct summary of the paper’s findings.

## 2. One-Dimensional Nonlinear Dynamic Transformation in Cross-Coupled Chaotic Systems

This section introduces a novel one-dimensional (1D) nonlinear dynamic transformation within cross-coupled chaotic systems. The system generates random numbers through an innovative algorithm grounded in the skew tent map, tailored specifically for FPGA implementation. To elucidate the chaotic behavior, we conduct an in-depth analysis of its nonlinear dynamics, employing various methods such as Lyapunov exponents (LEs), bifurcation diagrams, phase portraits, and entropy measures.

### 2.1. Basic Model

In this study, we utilize the skew tent map as the core mathematical framework. The skew tent map, defined by its two distinct piecewise linear segments, offers simplicity and computational efficiency, making it highly applicable to fields such as randomness generation, cryptography, and various security-related applications. A notable feature of our model is its flexibility for further refinement; the skew tent map can be replaced with other chaotic maps, presenting an intriguing avenue for future research aimed at boosting performance. The mathematical formulation of the Tent function is as follows:

(1) $$x_{n+1}=f_{p}\left(x_{n}\right)=\left\{\begin{array}{cc} \frac{x_{n}}{p}, & \;\mathrm{if}\;0\leq x<p\\ \frac{1-x_{n}}{1-p}, & \;\mathrm{if}\;p\leq x\leq 1 \end{array}\right.$$

The parameter *p* is constrained within the range (0,1). While the addition of simple coupling and associated subsequent processing functions to tilted tent diagrams can generate random numbers, their shortcomings in terms of nonlinear dynamic behavior, input range restriction, and periodicity, as well as the complexity of division, limit their application to FPGAs and other embedded scenarios [19]. To overcome these limitations, we propose an innovative dynamic algorithm system designed to enhance randomness generation capabilities, specifically optimized for FPGA performance.

Our methodology introduces a dynamic system designed to construct a one-dimensional nonlinear dynamic transformation coupled chaotic map, designated as 1d-dtts. This map is characterized by two interdependent branches, where the output of one branch serves as the input for the other, subsequently feeding back into its own input. The mathematical representation is articulated as follows:

(2) $$\left\{\begin{array}{c} p_{n+1}=\left\{\begin{array}{cc} \frac{p_{n}}{q}, & \mathrm{if}\;0<p_{n}<q\\ \frac{1-P_{n}}{1-q}, & \mathrm{if}\;q<p_{n}<1 \end{array}\right.\\ x_{n+1}=\left\{\begin{array}{cc} \left[\frac{\left(x_{n}⊕P_{n}\right)\times 2^{{\mathrm{num}}_{1}}}{z}+p_{n}^{\prime }\right]\;\mathrm{mod}\;1, & \mathrm{if}\;0<x_{n}<p_{n}\\ \left[\frac{\left(1-\left(x_{n}⊕p_{n}^{\prime }\right)\right)}{\left(1-z\right)\times 2^{{\mathrm{num}}_{2}}}+p_{n}\right]\;\mathrm{mod}\;1, & \mathrm{if}\;p_{n}<x_{n}<1 \end{array}\right. \end{array}\right.$$

In this set of equations, *z* and *q* are controlled parameters, both within the range of (0,1). ${\mathrm{num}}_{1}$ and ${\mathrm{num}}_{2}$ are dynamic transformation factors. In practical computations, ${\mathrm{num}}_{1}$ is specifically determined by the last *n* digits of *p*. In our FPGA explanation, we will analyze in detail the selection of *n* because these parameters primarily contribute to the randomness in FPGA computations. ${\mathrm{num}}_{1}$ and ${\mathrm{num}}_{2}$ are related as follows:

(3) $${\mathrm{num}}_{2}=2^{n}-{\mathrm{num}}_{1}$$

Moreover, we further improve randomness by adopting a coupled exchange method. Here, *p* and *x* have coupled exchange counterparts, $p^{\prime }$ and $x^{\prime }$. After coupling, we further optimize the algorithm in FPGA to make the efficiency of coupled computation superior to that of independently computing twice, effectively increasing throughput. We will elaborate on this in subsequent discussions. Hence, the final random number generated in a single computation is actually $x=\mathrm{concat}(x_{1},x_{1}^{\prime })$.

The multi-depth characteristic of the algorithm is reflected in the calculation of *z* in the update logic of *x*, while the dynamic transformation characteristic is manifested through the participation of different *p* and *x* values in each iteration.

### 2.2. Lyapunov Exponent

The Lyapunov exponent quantifies the mean exponential rate at which adjacent trajectories in phase space diverge, serving as a critical metric for assessing chaotic dynamics. The presence of multiple positive Lyapunov exponents indicates the intricate nature of a chaotic system’s behavior. Specifically, a system is deemed chaotic if it possesses two or more positive Lyapunov exponents and displays bounded, non-divergent trajectories. For a one-dimensional dynamical system characterized by $x_{i+1}=f\left(x_{i}\right)$, the analytical determination proceeds as follows:

(4) $$\lambda =\lim_{n\to \infty }\frac{1}{n}\sum_{i=0}^{n-1}\ln\left|f^{\prime }\left(x_{i}\right)\right|.$$

However, due to the nonlinearity of our equation and in accordance with the original definition, we calculate it by defining the derivative, as shown in the following equation:

(5) $$\lambda =\lim_{n\to \infty }\lim_{\Delta d\to 0}\frac{1}{n}\sum_{i=0}^{n+1}\ln\left|\frac{f\left(x_{i}+\Delta d\right)-f\left(x_{i}\right)}{\Delta d}\right|$$

**Figure 1:**
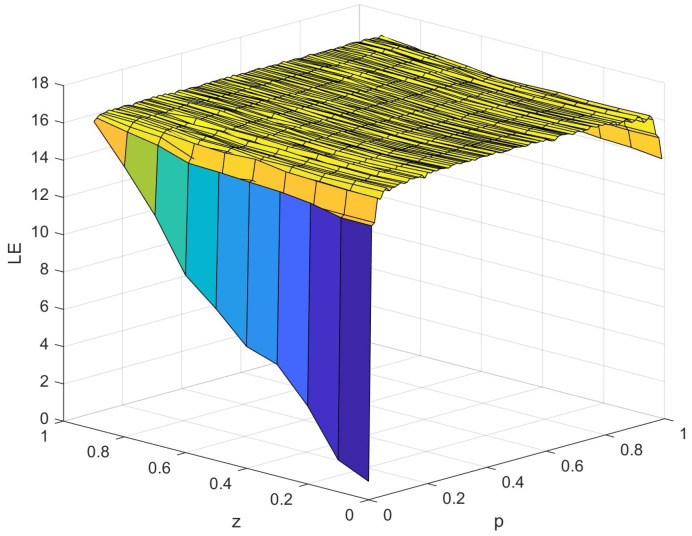
Lyapunov exponents of the MDDT system (3D), where the color of the figure represents the value of the angle.

Figure 1 presents the Lyapunov exponents (LEs) of the MDDT system. Since the MDDT system has two parameters, *q* and *z*, a three-dimensional plot is used. Evidently, the proposed system exhibits Lyapunov exponents greater than 0 for any combination of the two parameters ranging from 0 to 1 and remains stably above 10 in most regions, indicating robust dynamical properties. By selecting *p* as the independent variable, we ensure a precise and reliable reflection of *x*’s variations. This choice is justified by the strong correlation and the ability to maintain the integrity of the mathematical evaluation.

### 2.3. Bifurcation and Trajectory

The chaotic behavior of such systems can be effectively demonstrated through bifurcation diagrams and chaotic trajectories. The bifurcation diagram depicts the system’s output values as a function of a varying parameter, while the chaotic trajectory illustrates the dynamic path of the system’s output over successive iterations, given a fixed initial value.

**Figure 2:**
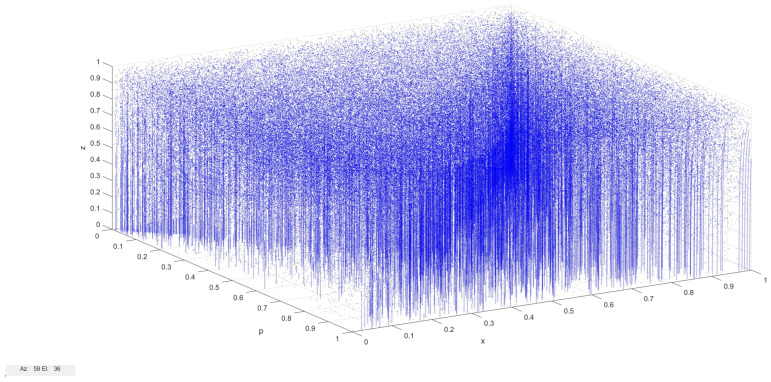
Bifurcation Diagram of the MDDT System (3D).

Figure 2 presents the bifurcation diagram of the MDDT system. Utilizing a three-dimensional representation, it showcases the random distribution of the output in response to changes in two control parameters, thereby indicating the extensive range of chaotic parameter choices. This extensive range underscores the inherent difficulty in influencing the randomness of the system.

**Figure 3:**
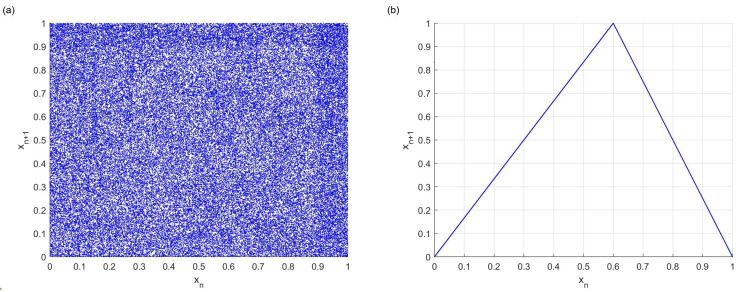
(**a**) Chaotic trajectories of the MDDT system (z=0.45,p=0.61); (**b**) chaotic trajectories of the skew tent system (p=0.499).

Figure 3a demonstrates the chaotic trajectories within our system, emphasizing both the uniform distribution and the superior randomness of these trajectories. Furthermore, a comparative analysis of the trajectory plots of MMDT and the skewed tent map, as illustrated in Figure 3b, is presented. The equation for the skewed tent map is as follows:

(6) $$f\left(x\right)=x_{n+1}=\left\{\begin{array}{cc} \frac{x_{n}}{p} & \mathrm{if}\;x\in R,x\in (0,p]\\ \frac{1-x_{n}}{1-p} & \mathrm{if}\;x\in R,x\in (p,1) \end{array}\right.$$

It clearly shows a distinct pattern: our trajectories are evenly spread throughout the entire space, exhibiting minimal correlations. In stark contrast, the trajectory plots of the skewed tent map are characterized by strong correlations and a lack of randomness.

Regarding the choice of z=0.45 and p=0.61 in Figure 3a, it is mainly based on the data analysis of Figure 1. It can be observed that when the *z* value is between 0.2 and 0.8, and the *p*-value is similarly between 0.2 and 0.8, the system has a significantly higher Lyapunov exponent, which suggests that the system exhibits a more strongly chaotic behavior. Therefore, the choice of specific values within this parameter range provides a certain degree of flexibility, while ensuring that the main dynamic properties of the model are not affected. Meanwhile, p=0.499 in Figure 3b is chosen to demonstrate the chaotic trajectory of the slanted tent algorithm itself. In the original slanted tent mapping, when the *p*-value is very close to 0.5, its chaotic properties can be demonstrated more clearly, thus proving its validity and characterization as a chaotic system [19]. This parameter choice not only focuses on theoretical validation but also facilitates the observation and comparison of behavioral differences between different chaotic mappings.

### 2.4. Entropy Analysis

To quantify the nonlinear dynamical characteristics of chaotic systems, various forms of entropy can be utilized. Sample entropy (SE), an approximate entropy measure, is extensively employed to evaluate the irregularity or complexity of time series data. A higher SE value indicates a greater degree of unpredictability in the dynamic behavior. Permutation entropy (PE), another significant metric, effectively gauges the complexity of chaotic time series, especially when dynamic and observational noise is present. PE employs permutations to evaluate the irregularity of reconstructed subsequences.

**Figure 4:**
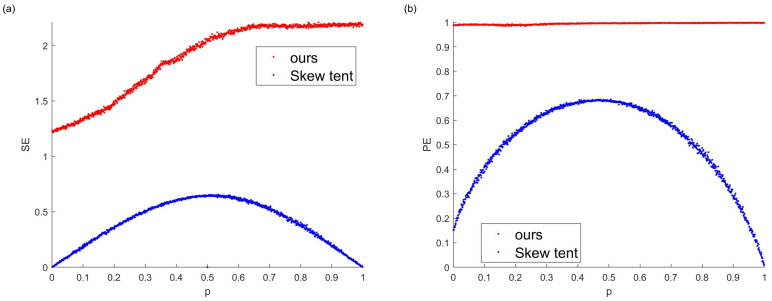
(**a**) Sample entropy: MDDT (z=0.45) vs. skew tent map; (**b**) permutation entropy: MDDT (z=0.45) vs. skew tent map.

Figure 4 provides a comparative analysis of the multiple deep-dynamic transformation (MDDT) map and skew tent chaotic map using different entropy measures. For consistency, the system parameters of all chaotic maps are aligned with those used in the prior comparative experiments, with the parameter *z* held constant and *p* varied within the MDDT system.

Figure 4a illustrates that the sample entropy (SE) produced by the multiple deep-dynamic transformation (MDDT) system surpasses that of the skew tent chaotic map, suggesting that the MDDT system generates a more complex time series. However, there is a significant reduction in the complexity of the MDDT time series around a value of 0.5, indicating that points near 0.5 should be avoided for optimal results.

Figure 4b reveals that the permutation entropy (PE) of the MDDT system approaches 1, while the PE of the skew tent system generally forms a tent-like shape. This comparison indicates that the MDDT system exhibits a higher degree of complexity.

## 3. Design of PRNG on FPGA

The hardware resource consumption is significantly influenced by the implementation method. For instance, the LUT resource consumption for implementing division is substantially higher than that for a shift approximation scheme. Simultaneously, the shift approximation scheme offers reduced latency. During computation, higher data representation precision enhances chaotic characteristics [22] and increases the output rate, albeit at the cost of greater hardware resource consumption. In this implementation, the computational depth of *X* is set to 24 bits, and the computational depth of *P* is set to 48 bits to balance hardware consumption and throughput.

Regarding the selection of the bit width, we conducted a series of experiments to determine the optimal computational bit width that produces sufficient randomness. The following table analyzes the tests on bit width with its final performance (In actual hardware algorithm design, arithmetic bit widths greater than 16 bits have been split into four groups for synchronized arithmetic, e.g., 24 bits are dynamically shifted in accordance with four groups of 6-bit wide sections):

From Table 1, it is clear that the 24-bit computational bit width and the [0:3] displacement range can satisfy both the randomness requirement and the clock constraints, which is the optimal solution based on the experimental data.

**Figure 5:**
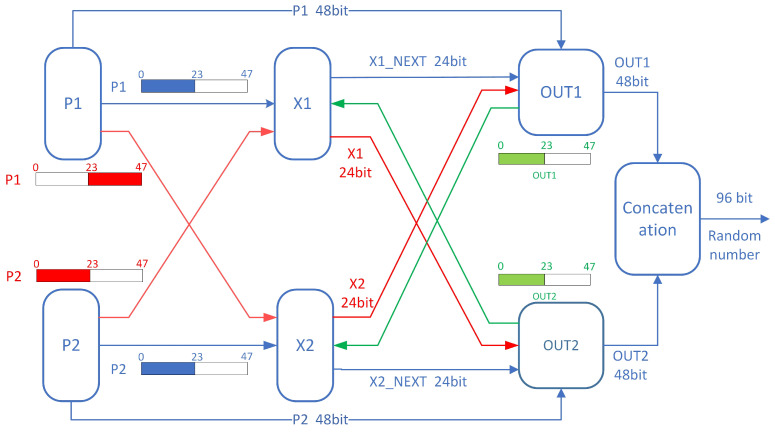
Schematic diagram of the data exchange of the random number part of the FPGA.

The initial parameters are set to $\left(X_{1},X_{2},P_{1},P_{2}\right)=\left(0\;\times \;6666,0\;\times \;9999,0\;\times \;3\mathrm{FFF},0\;\times \;F3\mathrm{FF}\right)$, with zeros padding the unused bits. To simplify complex arithmetic operations and reduce latency, fixed division in the intricate *P* update logic is replaced by an approximate shift implementation. Since the *X* update logic involves subtraction, addition, and dynamic shifting, it will consume a lot of combinational logic on the FPGA, especially the dynamic shifting in the *X* update logic, which is the most time-consuming step, and in order to satisfy the timing constraints at higher frequencies, it often requires multiple clock cycles to complete, and in order to take into account the throughput of the random number generation, it is often necessary to make the arithmetic operation complete within a single clock cycle, thus Therefore, relevant optimizations are necessary. Therefore, the *X* update logic is segmented to ensure normal operation at high frequencies. To maintain effective chaotic performance with smaller bit-width segmented operations, this implementation employs two similar *X* calculation modules for coupled calculations. The *X* update implementation introduces cross-coupling concerning *P*, with the output from the opposite side of the previous iteration being used as the new *X* input coupling. Additionally, cross-coupling concerning *X* itself is introduced in the output processing splicing part, as shown in Figure 5.

This modification of splitting and re-combining the x-module arithmetic process is actually an optimization of the dynamic shifts in the original MDDT algorithm. The x-module part of the original MDDT algorithm shows good nonlinear characteristics in the algorithmic analysis when only one dynamic shift is used. The FPGA hardware implementation version adopts splitting the *X* into four segments, and then performs the dynamic shift operation synchronously, and then splices them together afterward, which is essentially a bit-width-reduced version of the nonlinear processing of the dynamic shifts of the *X*-part of the original MDDT algorithm by combining four of the original MDDT algorithm’s *X*-part into one x-module. In essence, the bit-width-reduced version of the four original MDDT algorithms for the nonlinear processing of the dynamic displacement of the *X* part is integrated into the optimized hardware version of the *X* module, i.e., the one nonlinear transformation of the *X* part of the original version is changed into four nonlinear transformations, and splicing is carried out after the completion of the dynamic displacement of the *X* part to keep the bit-width of the *X* module as 24 bits, and then continue the rest of the original MDDT algorithm. Obviously, this optimization not only does not weaken the performance of the FPGA hardware implementation version of the random number generator, but also reduces the actual operational bit-width, improves the operational efficiency through the use of synchronous shifts, reduces resource consumption and clock delay, increases the maximum clock frequency under the FPGA design timing constraints, and enhances the performance of the random numbers it generates. This is demonstrated in the following output random number test section, where the random numbers generated by the FPGA hardware version of the random number generator successfully pass the NIST test and other tests, with good performance in all indicators.

### 3.1. Calculation and Update Logic of X

**Figure 6:**
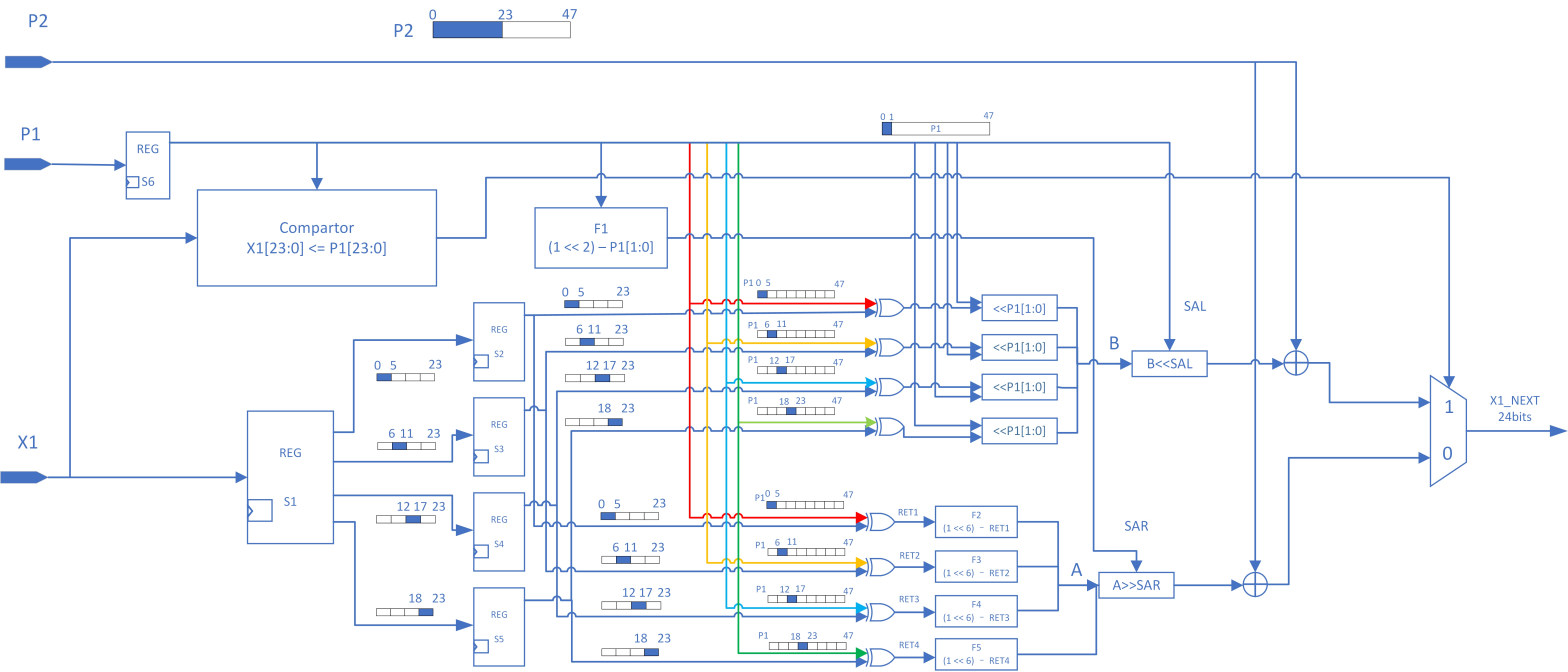
Hardware design structure of the *X* update logic implemented on FPGA.

The two update modules, $X_{1}$ and $X_{2}$, operate simultaneously. During the initial initialization, the inputs are the initial seeds $X_{1}$ and $X_{2}$. During normal operation, the inputs are the lower 24 bits of the opposite side’s output, as illustrated in Figure 5. The *P* update modules, $P_{1}$ and $P_{2}$, are also required as inputs to participate in the calculations of $X_{2}$ and $X_{1}$ in a cross-coupled manner, as shown in Figure 6. The lower 2 bits of *P* are also necessary for calculating dynamic shifts. To reduce latency, the complex conditional subtraction and dynamic shift in the *X* update are divided into four 6-bit wide parts for parallel processing. Upon completion, these parts are concatenated into a 24-bit value in a fixed order, performing a dynamic shift first, followed by a 24-bit wide XOR with the coupled *P*.

The formula for the shift width is shown in Equation (7):

(7) $$SAR=\left(1<<2\right)-P_{\left[1:0\right]}$$

where SAR represents the number of shift bits. Since SAR uses unsigned integers, the range of SAR is [1,4]. This range is chosen considering that the calculation width of *X* after splitting is 6 bits, and the maximum bit shift cannot exceed the width of a single part after splitting.

#### 3.1.1. Calculation and Update Logic of $X_{1}$

The update logic for $X_{1}$ is a crucial part of our FPGA implementation. This algorithm, described in Algorithm 1, takes the current value of $X_{1}$ along with the parameters P1 and P2 as inputs and produces the next value of $X_{1}$. The process involves bit manipulation operations and conditional logic based on the relationship between $X_{1}$ and P1.
**Algorithm 1:** Update logic for $X_{1}$ on FPGA
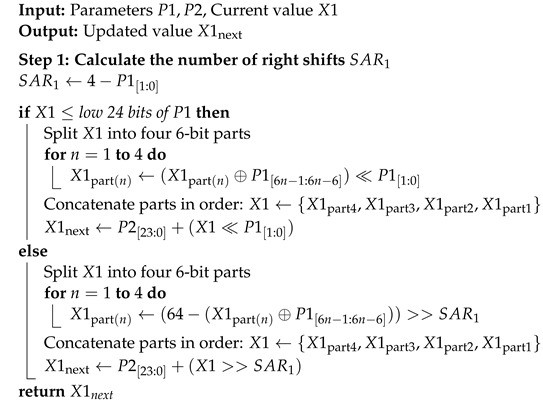


#### 3.1.2. Calculation and Update Logic of $X_{2}$

Similar to the update logic for $X_{1}$, the algorithm for updating $X_{2}$ is another key element in our FPGA design. Algorithm 2 outlines this process, which takes the current value of $X_{2}$ and the parameters P1 and P2 as inputs to calculate the next value of $X_{2}$. The algorithm uses similar bit manipulation techniques and conditional logic as the $X_{1}$ update, but with different parameter interactions.
**Algorithm 2:** Update logic for $X_{2}$ on FPGA
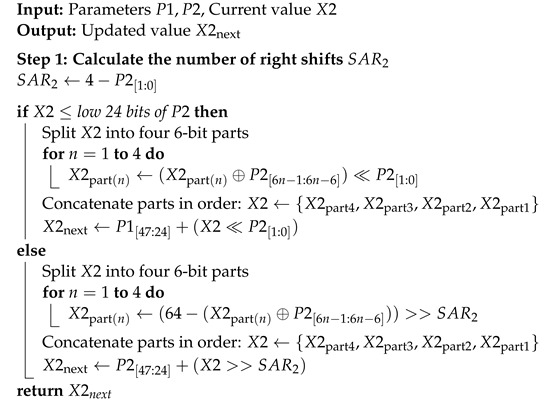


### 3.2. Implementation of P Update Logic on FPGA

**Figure 7:**
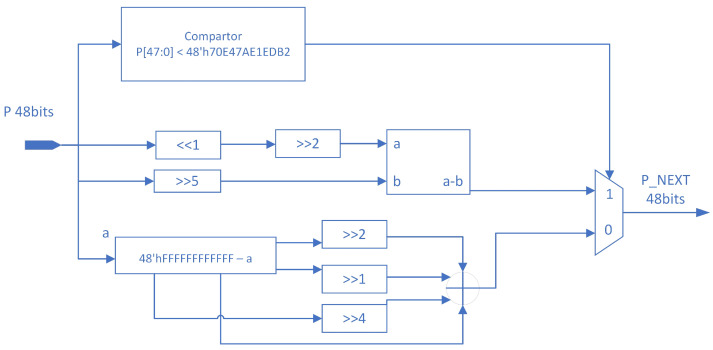
Implementation of *P* update logic on FPGA.

*P* simulates the standard skew tent map update logic but carefully selects appropriate divisors. By using shifts instead of division, resource usage and latency are reduced. The implementation logic is illustrated in Figure 7.

#### *P* Update

The update logic for the two *P* modules is identical, hence, P(n) is used in the calculation formulas, where *n* ranges from 1 to 2.

If P(n) is less than 45% of the maximum value that it can represent, a skew tent-like logic is applied to calculate *P*, specifically multiplying P(n) by 2.46875. This approximates division by 0.45 using shifts, as shown in Figure 7.

Conversely, if P(n) is greater than 45% of the maximum value it can represent, a skew tent-like logic is applied to calculate *P*. This process involves subtracting P(n) from the maximum value represented by 48 bits and then multiplying the result by 1.8125 using shifts. This technique approximates division by 0.55, thereby effectively reducing resource consumption and latency. The number 1.8125 is chosen to simulate in the hardware design an effect similar to that of dividing by 0.55 (i.e., the result of 1−0.45), essentially using multiplication instead of division. Performing the division operation directly in hardware typically involves higher complexity and cost. For efficiency and practicality, the effect of division is approximated by a combination of displacements. The selection process is based on careful considerations. We first consider the combination of displacements to achieve the desired computational result, i.e., to simulate the operation of dividing by 0.55. It is found that multiplying by 1.8125 (which is actually a combination of 1 [*P* itself] + 0.25 [*P* shifted 2 bits to the right] + 0.5 [*P* shifted 1 bit to the right] + 0.0625 [*P* shifted four bits to the right]) better approximates this effect. Although this method has some errors compared to the direct division operation, this error is acceptable in most application scenarios.

### 3.3. Calculation of Final 96-Bit Random Number Output on FPGA

The final 96-bit random number output is derived by concatenating two 48-bit outputs, out1 and out2, as shown in Equation (8):

(8) Result={out1,out2}

Both out1 and out2 are obtained by XORing the corresponding *X* and *p* values. The following sections describe the logic for calculating out1 and out2.

#### 3.3.1. Calculation Logic for out1

**Figure 8:**
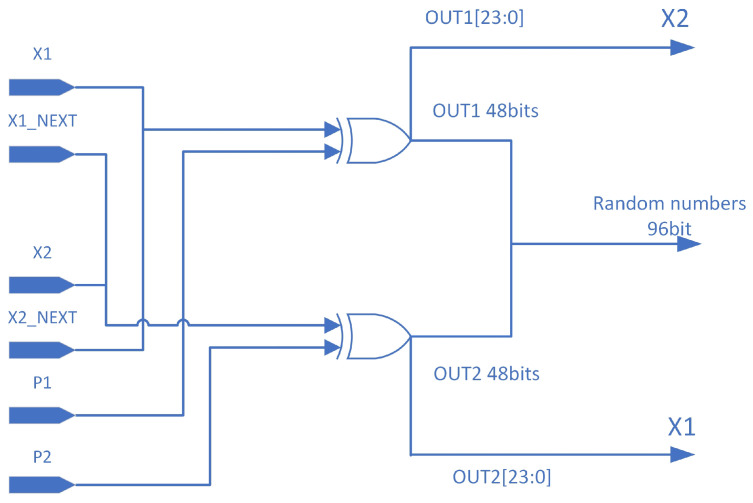
Calculation logic for out1 and out2.

As shown in Figure 8, the 24-bit $X_{2}$ is concatenated with the computed $X1_{\mathrm{next}}$, and the resulting 48-bit value is XORed with $P_{1}$ to obtain out1. The calculation formula is given by Equation (9):

(9) $$out1=\{X1,X2_{\mathrm{next}}\}⊕P1$$

#### 3.3.2. Calculation Logic for out2

As shown in Figure 8, the 24-bit $X_{1}$ is concatenated with the computed $X2_{\mathrm{next}}$, and the resulting 48-bit value is XORed with $P_{2}$ to obtain out2. The calculation formula is given by Equation (10):

(10) $$out2=\{X2,X1_{\mathrm{next}}\}⊕P2$$

Through the above series of operations, the FPGA implementation achieves efficient utilization of all computation results under a smaller bit-width calculation premise. The high-speed self-oscillating property of *P* and the multi-module coupling maintain a high unpredictability of the generated sequence, enabling the output of a 96-bit random number per clock cycle with only 24-bit wide *X* and 48-bit wide *P* calculations.

## 4. Experimental Result

This section outlines the implementation of the PRNG on the Xilinx Kintex-7 xc7k325tffg900-2 evaluation kit and assesses its throughput based on the timing report. It can be confirmed that the system is stable, as both the worst negative slack (WNS) and total negative slack (TNS) are positive. Table 2 illustrates the WNS for the three most critical paths at a frequency of 150 MHz, thereby demonstrating that the system is stable at this frequency.

Our multiple deep-dynamic transformation (MDDT) random number system begins with four seed values: $X_{1}$, $X_{2}$, $P_{1}$, and $P_{2}$. These seeds undergo deep-dynamic transformations synchronized with the system clock. The 96-bit output is generated in just one clock cycle per iteration, resulting in a throughput of 14.4 Gbps at a core frequency of 150 MHz. Table 3 presents the FPGA hardware consumption of our system, which utilizes only 0.67% of the FPGA’s resources while achieving high throughput.

The clock frequency in the FPGA implementations of all the algorithms involved in the comparison does not reach the limit of the FPGA development boards used, and the hardware resources consumed do not reach the limit of the FPGA development boards; at the same time, the random number generation algorithms and hardware implementations, once determined, the demand for hardware resources (e.g., Slices, LUTs, FFs, DSPs, etc.) is fixed, so the algorithms’ consumption of hardware resources is determined by the algorithm design itself, independent of the specific development board. Therefore, the consumption of hardware resources by the algorithm is determined by the algorithm design itself, independent of the specific development board, the main purpose of this comparison is to highlight the performance difference of the algorithm itself rather than the difference in hardware.

Our design offers several clear advantages. Firstly, the MDDT operates as a discrete chaotic map, capable of producing complex outputs without requiring post-processing, thus conserving significant hardware resources. Secondly, our design employs simple shift-add-XOR logic, facilitating normal operation at high frequencies while minimizing resource usage and avoiding the need for DSPs.

**Figure 9:**
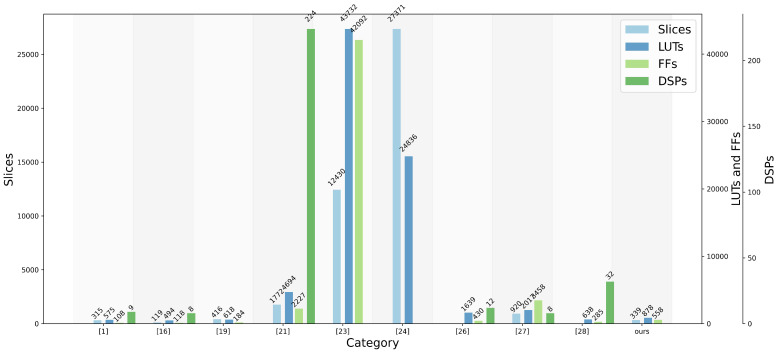
Hardware resource utilization for various random number generators [1,16,19,21,23,24,26,27,28].

The FPGA resource utilization comparison across different systems is illustrated in Figure 9. By utilizing straightforward shift operations, our proposed PRNG achieves high-frequency operation while consuming only 878 LUTs and 558 flip-flops (FFs). Like our implementation, studies such as [19,24,25] do not provide details on DSP consumption.

Table 4 presents a comprehensive comparison of our PRNG with other existing PRNGs. While the SPCS PRNG [24] and P3DS PRNG [26] operate at higher frequencies, they exhibit significantly lower throughput compared to ours. Although the TL-LCHM PRNG [28] operates at a similar frequency, our throughput surpasses it by more than 1.5 times. The BM PRNG [1] and Lorenz+Lu PRNG [16], despite focusing on low resource consumption, lag in throughput. Remarkably, our design, devoid of DSP usage, achieves higher overall generation speed and random number generation speed per LUT compared to the BM PRNG [1] and Lorenz+Lu PRNG [16]. Specifically, our overall generation speed and per LUT random number generation speeds are 7.67 times and 4.3 times that of the Lorenz+Lu PRNG [16], respectively. Compared to the 5D-FFRK random number generator [27], our design exhibits a 1.6-fold increase in output speed per unit frequency while utilizing only 36.8% of the Slices, 43.5% of the LUTs, and 16.1% of the FFs.

Power analysis was conducted using the Xilinx® Power Estimator (XPE)-2019.2.2. The total power consumption of the MDDT is estimated to be 218 mW at a throughput of 14.4 Gbps, resulting in an energy efficiency of approximately 15.28 pJ/b. This efficiency is notably superior to the 61.964 pJ/b reported for the TL-COTDCM PRNG [21] and the 34.2 pJ/b reported for the TL-LCHM [28].

## 5. Evaluation of Randomness

### 5.1. Histogram

**Figure 10:**
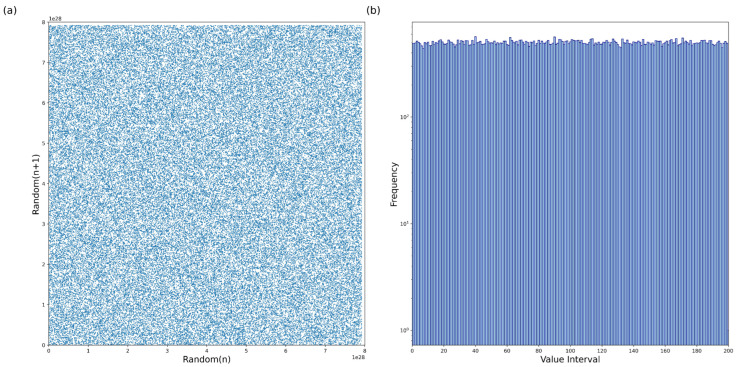
(**a**) $Random_{n}-Random_{n+1}$ trajectory points. (**b**) Histogram of random bitstreams.

To evaluate the statistical attributes of random sequences generated, histograms are employed. A PRNG is deemed statistically excellent if the standard deviation in the histogram distribution of the random numbers is very small. We analyzed a chaotic sequence of 96 bits with a length of 1,000,000 to determine its distribution characteristics. As depicted in Figure 10a, the distribution of these sequences corroborates our mathematical assessments. Figure 10b illustrates the histogram of the output bits, where the range of 96 bits is segmented into 200 equal parts on the x-axis. This histogram displays a notably irregular distribution, suggestive of an absence of discernible patterns.

### 5.2. TestU01 Evaluation

TestU01 is a widely utilized test suite providing an extensive range of tests for evaluating random bit generators. This suite includes generic and versatile statistical testing tools [28]. For our evaluation, we employed two specific test batteries: the Rabbit and Alphabit tests. The Rabbit test battery comprises 39 subtests, while the Alphabit test battery consists of 17 subtests, each applied to sequences of $2^{32}$ bits. Additionally, for each 24-fold increase in sequence length, an additional Rabbit test is required for comprehensive acceptance testing.

Table 5 displays the results of the battery tests, demonstrating that all tests pass even for sequences as long as $2^{32}$ bits. This is significantly longer than the $2^{28}$-bit length reported in [21].

### 5.3. NIST Statistical Test Suite

The NIST SP800-22 test suite comprises multiple statistical tests designed to evaluate the randomness of binary sequences [29]. By scrutinizing various non-randomness patterns, it serves as a benchmark for detecting deviations from randomness. Typically, a significance level of α=0.01 is employed, with *p*-values greater than or equal to 0.01 considered acceptable. The randomness of the test data is confirmed when the proportion falls within the confidence interval of [0.9805607,0.9994392]. The outcomes of the NIST statistical tests are outlined in Table 6, where “*” signifies the test’s average result. For a significance level of 0.01 and a sample size of 1000 sequences, each statistical test requires a minimum passing rate of around 980. Similarly, for a sample size of 618 binary sequences, this threshold is approximately 606. Notably, our pseudo-random number generator (PRNG) successfully cleared all NIST SP800-22 statistical tests.

### 5.4. Diehard Statistical Test Suite

The diehard test suite, composed of 15 independent statistical tests, is utilized to analyze files consisting of millions of 32-bit integers [30]. These tests generate a series of statistical *p*-values, and a uniform distribution of these values between 0 and 1 indicates randomness. To enhance result reliability, it is advisable to conduct the tests multiple times using different sets of integers. For our evaluation, we generated a 500 MB binary file employing a method akin to TestU01. As illustrated in Table 7, all diehard tests were successfully passed, suggesting that our output sequence exhibits commendable randomness.

## 6. Conclusions

We engineered a PRNG employing MDDT of chaotic systems, specifically implemented on an FPGA. This design is aimed at achieving high throughput, exceptional randomness, and efficient utilization of hardware resources. The complex nonlinear dynamics intrinsic to chaotic systems confer a substantial degree of randomness to the generated sequences. Experimental data demonstrate that substituting conventional division operations with shift and slice operations markedly improves hardware utilization efficiency. This PRNG is capable of generating up to 14.4Gbps of random output at a maximum stable clock frequency of 150MHz, eliminating the need for post-processing. Additionally, it consumes less than 0.7% of FPGA resources, even when producing a 96-bit stream. The generated output bits have undergone extensive evaluation through a multitude of established statistical tests, consistently demonstrating a high degree of randomness. Consequently, this PRNG is well-suited for contemporary industrial applications, including confidential computing and image encryption.

## Figures and Tables

**Table 1:** Calculated bit width of *x* and dynamic displacement width range selection and its result analysis.

Computational Bit Width\Dynamic Displacement Range	8 Bits	16 Bits	24 Bits	48 Bits	96 Bits	128 Bits
**[0:1]**	Fails NIST tests	Fails NIST tests	Fails NIST tests	Fails NIST tests	Fails NIST tests	Fails NIST tests
**[0:3]**	Meets clock, fails throughput speed	Fails NIST tests	Meets clock, randomness, high throughput	High delay, fails clock	Fails NIST tests	Fails NIST tests
**[0:7]**	Shifts exceed width	Fails NIST tests	Shifts exceed width	Fails NIST tests	High delay, fails clock	Fails NIST tests
**[0:15]**	Shifts exceed width	Shifts exceed width	Shifts exceed width	Shifts exceed width	High delay, fails clock	Fails NIST tests

**Table 2:** WNS of the three most critical paths at 150 MHz.

Name	Slack (ms)	Total Delay (ms)	Details
**PATH1**	0.102	6.437	1.064 (Logic) + 5.267 (Net)
**PATH2**	0.112	6.436	1.169 (Logic) + 5.373 (Net)
**PATH3**	0.161	6.455	1.278 (Logic) + 5.167 (Net)

**Table 3:** Comparison of entropy sources and FPGA implementations.

Refs.	Types	Entropy Source	FPGA Chip	System Type	Throughput (Mb/s)	Max. Clock Frequency (MHz)	Post Processing
[1]	Pseudo	BM	Spartan 3E	Discrete	7.38	36.9	XOR
[16]	Pseudo	Lorenz+Lu	Virtex-V	Continuous	1875.58	78.149	Reconfigured
[19]	Pseudo	3D multi-scroll	Spartan-3E	Discrete	289	37.89	-
[21]	Pseudo	TL-COTDCM	Kintex-7	Discrete	10,283.52	120.51	-
[22]	TRUE	MCS+LM	Kintex-7	Continuous	0.125	59.492	XOR
[23]	Pseudo	DE	Zybo Z7–20	Discrete	169.31	105.82	-
[24]	TRUE	SPCS	Virtex-6	Continuous	58.7	293.82	XOR
[25]	Pseudo	FWMHS+BM	ZYNQ-XC7Z020	Continuous	62.5	135.04	XOR
[26]	TRUE	P3DS	Virtex-6	Continuous	464.89	464.89	XOR
[27]	Pseudo	5D-FFRK	Zynq-7000	Discrete	6780	113	-
[28]	Pseudo	TL-LCHM	Kintex-7	Discrete	9480	158	-
ours	Pseudo	MDDT	Kintex-7	Discrete	14,400	150	-

**Table 4:** FPGA resource utilization for the proposed PRNG.

Resource Type	Look-Up Tables (LUTs)	Flip-Flops (FFs)	Slices
**Utilization Count**	878	558	339
**Percentage Usage**	0.43%	0.14%	0.67%

**Table 5:** Results of battery tests for various lengths of random bits.

Sequence Length	Rabbit Test Results	Alphabit Test Results
$2^{28}$	40/40	17/17
$2^{29}$	40/40	17/17
$2^{30}$	40/40	17/17
$2^{31}$	40/40	17/17
$2^{32}$	40/40	17/17

**Table 6:** Results of NIST SP800-22 Test, N = 1000 sequences of length 1 Mb.

NIST Test	*p*-Value	Passing Proportion
Frequency	0.220159	992/1000
Block frequency	0.128132	994/1000
Cumulative sums *	0.567838	992/1000
Runs	0.574903	991/1000
Longest run	0.205531	990/1000
Rank	0.991468	993/1000
FFT	0.967382	990/1000
Non-overlapping template *	0.504044	990/1000
Overlapping template	0.234373	987/1000
Universal	0.607993	991/1000
Approximate entropy	0.564639	993/1000
Random excursion *	0.586345	612/618
Random excursion variant *	0.209342	614/618
Serial *	0.0711915	993/1000
Linear complexity	0.214439	990/1000

**Table 7:** Results of diehard tests for the proposed PRNG, sequences of 500 M BITS.

Diehard Test	*p*-Value	Result
Birthdays	0.71158168	PASSED
Operm5	0.09298933	PASSED
Rank 6×8	0.6927688	PASSED
Rank 32×32	0.39556777	PASSED
Bitstream	0.10464763	PASSED
OPSO	0.9359973	PASSED
DNA	0.46640039	PASSED
Count 1s string	0.02640681	PASSED
Count 1s byte	0.02178915	PASSED
Parking lot	0.6565555	PASSED
2d sphere	0.7759774	PASSED
3d sphere	0.96829087	PASSED
Squeeze	0.95464699	PASSED
Sums	0.04541152	PASSED
Runs up	0.81875048	PASSED
Runs down	0.59606009	PASSED
Craps	0.67008209	PASSED

## Data Availability

The original contributions presented in the study are included in the article, further inquiries can be directed to the corresponding author.
